# Paper-Based Devices for Capturing Exosomes and Exosomal Nucleic Acids From Biological Samples

**DOI:** 10.3389/fbioe.2022.836082

**Published:** 2022-04-12

**Authors:** Chi-Hung Lai, Chih-Ling Lee, Cao-An Vu, Van-Truc Vu, Yao-Hung Tsai, Wen-Yih Chen, Chao-Min Cheng

**Affiliations:** ^1^ Department of Chemical and Materials Engineering, National Central University, Taoyuan, Taiwan; ^2^ Institute of Biomedical Engineering, National Tsing Hua University, Hsinchu, Taiwan

**Keywords:** paper-based device, exosome, exosomal miRNA, nucleic acid extraction, immunoassay, plasma, wound fluid, colorimetric sensing

## Abstract

Exosomes, nanovesicles derived from cells, contain a variety of biomolecules that can be considered biomarkers for disease diagnosis, including microRNAs (miRNAs). Given knowledge and demand, inexpensive, robust, and easy-to-use tools that are compatible with downstream nucleic acid detection should be developed to replace traditional methodologies for point-of-care testing (POCT) applications. This study deploys a paper-based extraction kit for exosome and exosomal miRNA analytical system with some quantifying methods to serve as an easy sample preparation for a possible POCT process. Exosomes concentrated from HCT116 cell cultures were arrested on paper-based immunoaffinity devices, which were produced by immobilizing anti-CD63 antibodies on Whatman filter paper, before being subjected to paper-based silica devices for nucleic acids to be trapped by silica nanoparticles adsorbed onto Whatman filter paper. Concentrations of captured exosomes were quantified by enzyme-linked immunosorbent assay (ELISA), demonstrating that paper-based immunoaffinity devices succeeded in capturing and determining exosome levels from cells cultured in both neutral and acidic microenvironments, whereas microRNA 21 (miR-21), a biomarker for various types of cancers and among the nucleic acids absorbed onto the silica devices, was determined by reverse transcription quantitative polymerase chain reaction (RT-qPCR) to prove that paper-based silica devices were capable of trapping exosomal nucleic acids. The developed paper-based kit and the devised procedure was successfully exploited to isolate exosomes and exosomal nucleic acids from different biological samples (platelet-poor plasma and lesion fluid) as clinical applications.

## Introduction

Multivesicular bodies (MVBs), which contain intraluminal vesicles (ILVs) formed by cell membrane invagination, are created intracellularly from the loading of biomolecules into ILVs by the cell of origin. The ILVs are then released from the cells (Akers et al., 2013; Mathieu et al., 2019) and ubiquitously distributed to the bladder, liver, spleen, bone, blood, heart, thyroid, lung, kidney, brain (Betzer et al., 2017; Abello et al., 2019; Faruqu et al., 2019; Rashid et al., 2019; Royo et al., 2019) after integrating with specific sites on the generating cell membrane. In 1889, the British surgeon Paget discovered that tumor cells easily metastasize in multiple -directions to tissues but can only thrive in a favorable microenvironment and proposed the “seed and soil hypothesis” (Liu et al., 2017). In 2015, Lyden et al. provided more comprehensive data indicating that tumor cells initially release exosomes that are transported to target organs and tissues and taken up by receptors (Hoshino et al., 2015). Exosomes (30–150 nm^10^), which are nanovesicles surrounded by a lipid bilayer, have recently been discovered to be released by various cells (Qiao et al., 2020; Yan and Jiang, 2020; Kumar et al., 2021; PinkyGupta et al., 2021) and carry an assortment of biological molecules, including proteins, lipids, nucleic acids (miRNA, mRNA, DNA), metabolites, etc. (Qing et al., 2018; Jeppesen et al., 2019; Sharma and Johnson, 2019; Zhang et al., 2019; Kalluri and LeBleu, 2020; Ocansey et al., 2020), which are delivered by cells of origin to specific receptor cells and are essential for the transmission of information between cells. Furthermore, exosomes are capable of influencing tumor growth by regulating immune function, promoting tumor angiogenesis and metastasis, and enhancing drug resistance of cancerous cells (Wan et al., 2018; Mashouri et al., 2019; Sharma and Johnson, 2019; Wortzel et al., 2019). Exosomes have been demonstrated to be highly associated with numerous cancers, including colorectal (Yan et al., 2017), gastric (Huang et al., 2020), liver (Li et al., 2017), lung (Park et al., 2017), pancreatic (Carmicheal et al., 2019), and prostate cancers (Lee et al., 2018). They have the potential for clinical applications and are considered a diagnostic marker to facilitate possible precision treatment.

MicroRNAs (miRNAs), one of the bio-species transported by exosomes, are a group of endogenously small noncoding RNAs that are responsible for posttranslational regulation in cells and play a very profound role in the development of pathology. miRNAs are vital biomarkers for disease diagnosis and prognosis because anomalous expression of miRNAs in cells and exosomes is highly correlated with a variety of human diseases. For example, it has been reported that certain miRNAs can be used as tumor oncogenes and suppressor genes to control cancerous cells and suppress tumor growth, respectively. Although several methodologies have been invented to facilitate precision disease diagnosis point-of-care testing (POCT or bedside testing, a medical analysis performed near the patients (Quesada-González and Merkoçi, 2018)) in developed nations, a number of challenges must be addressed prior to their appropriate application in clinical practice. These obstacles are sample purification difficulties and the need for expensive equipment that restricts rapid and broadly available testing, which is even more difficult to overcome in developing or underdeveloped countries. Therefore, an appropriately simple and robust approach for the rapid extraction of nucleic acids that can be integrated into POCT would significantly impact precision and companion disease diagnosis.

**FIGURE 1:**
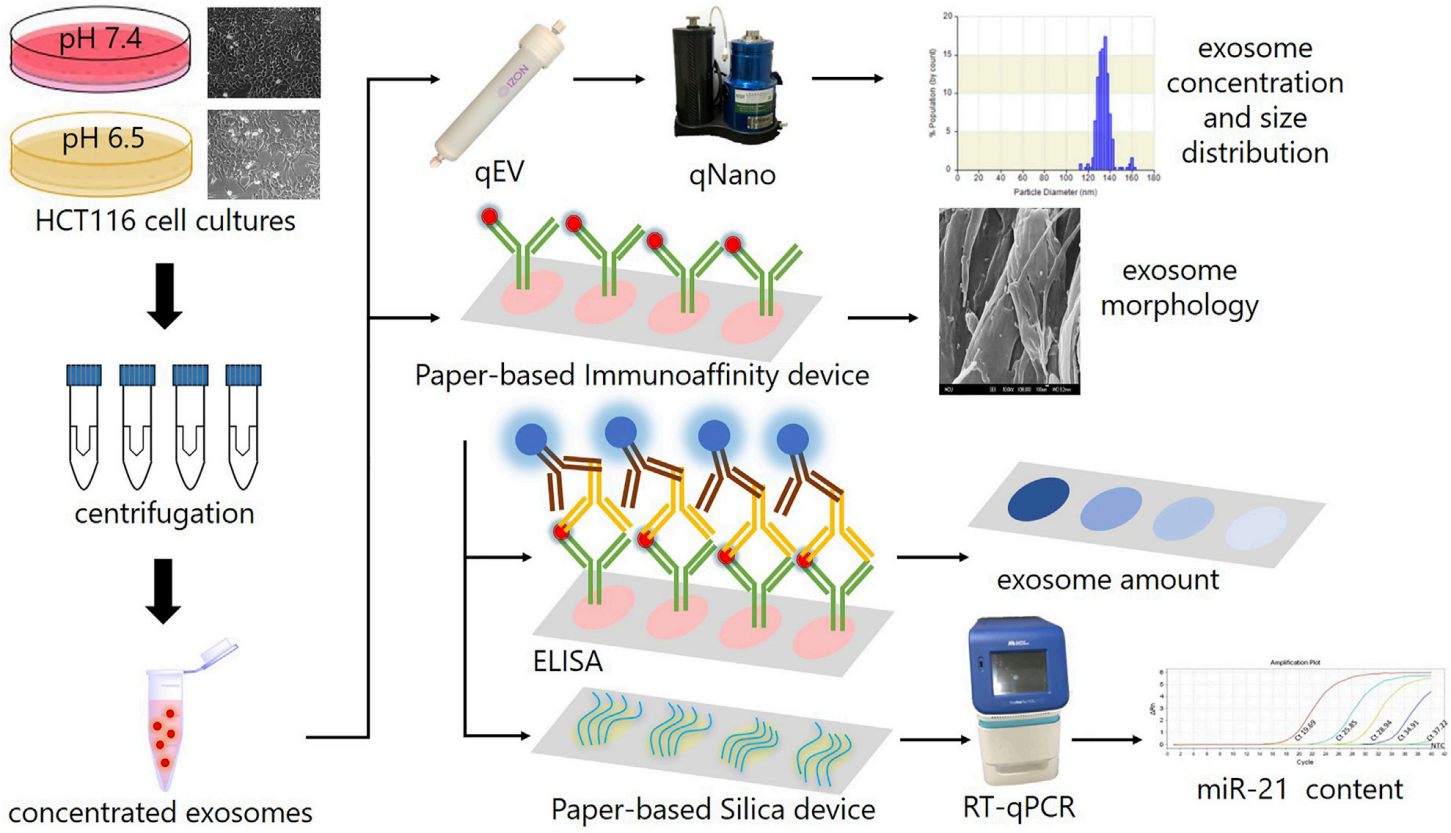
The scheme describes the experimental design in this study. HCT116 cells were cultured in various microenvironments (pH 7.4 and 6.5), characterized by microscope, ultrafiltrated to collect concentrated exosomes, which were then analyzed by a qNano instrument to determine their concentration and size distribution, and subjected to paper-based immunoaffinity devices for exosome capture. The morphology of captured exosomes was subsequently characterized by SEM and their amount was quantified by ELISA. Eventually, exosomal nucleic acids were absorbed by paper-based silica devices for miR-21 quantification by RT-qPCR.

Lab-on-chip (LOC), a description of miniaturized devices at millimeter-to-centimeter scales that integrate single or multiple functions of a laboratory into a chip (Sengupta and Hussain, 2019), is one of two major types of POCT devices (Romao et al., 2017). A category of LOC devices, paper-based analytical devices (PADs), have recently emerged as promising candidates for POCT because of their low cost, simplicity, recyclability, and disposability by incineration (Smith et al., 2018; Hristov et al., 2019). In comparison with conventional instruments, LOC is not only more portable but also can be operated without external power resources (Smith et al., 2018; Hristov et al., 2019). More importantly, it has been demonstrated to potentially fulfill the ASSURED criteria (affordable, sensitive, specific, user-friendly, rapid and robust, equipment-free, and deliverable to end users) proposed by the World Health Organization (WHO) for the development of POCT applications in areas with limited resources (Hu et al., 2014; Smith et al., 2018). Exploiting the rapid growth and recent advances in paper-based technologies for POCT, especially in nucleic acids and immunoassays (Choi et al., 2017; Zhu et al., 2019; Lee et al., 2020; Mahmoudi et al., 2020), this research deployed a novel and easy-to-use technique for sample preparation of exosomal miRNA as biomarker for liquid biopsy by designing a paper-based system, including paper-based immunoaffinity and paper-based silica devices, to capture exosomes derived from HCT116 cells cultured in varied microenvironments and nucleic acids carried by the harvested exosomes. The cells were initially observed by microscopy and the culture media undergoing an ultrafiltration step to concentrate the exosomes and subsequently analyzed by qNano. The paper-based immunoaffinity devices were then employed to capture concentrated exosomes before being undergone lysis buffer and silica-coated papers to trap nucleic acids. The captured exosomes were quantified by enzyme-linked immunosorbent assay (ELISA) and characterized by field-emission scanning electron microscopy (FE-SEM) whereas microRNA 21 (miR-21), a representative target among the exosomal nucleic acids trapped by paper-based silica devices, was determined by quantitative reverse transcription polymerase chain reaction (RT-qPCR) to examine the operability of the developed paper-based system. (Figure 1). This system was finally exploited to isolate exosomes and exosomal nucleic acids from various clinical samples (plasma and wound fluid) as a practical application of sample preparation.

## Materials and Methods

### Cell Cultures for Capturing Exosome

HCT116 cancer cells (human colorectal cancer cells) were provided by the Laboratory of Molecular Diagnostics and Therapeutics, Department of Biomedical Sciences and Engineering, National Central University (NCU), Taiwan. The cells were cultured in McCoy’s 5A (modified) medium containing 1% penicillin/streptomycin double antibiotic solution and 10% fetal bovine serum (all from Thermo Fisher Scientific, United States ) and incubated at 37°C with 5% CO_2_ for 3 days. To create different microenvironments, we adjusted the pH value from 7.4 to 6.5 by adding 1 M HCl (ECHO Chemical, Taiwan). The cell morphology was captured at ×100 magnification by a microscopy instrument.

### Concentrating HCT116 Cell-Derived Exosomes

Media were collected and centrifuged at 1,200 g for 15 min to remove cells and unwanted contaminants before transferring the supernatant to new containers for centrifugation at 8,000 g in 30 min to remove cell debris. The media were again centrifuged a third time at 10,000 g for 30 min in a 10-kDa PES vivaspin concentrate tube (GE Healthcare, UK). The last centrifugation to purify the contents was completed at 10,000 g for 30 min after adding 3 ml PBS. This final suspension was then 1) analyzed by a qNano instrument and 2) used as materials for exosome extraction by paper-based immunoaffinity devices.

### Analyzing the Size Distribution of Concentrated Exosomes by qNano

The size distribution of the exosomes captured from cell cultures was analyzed by a qNano Particle Analyzer (IZON, Science New Zealand) after pretreatment with a qEV size exclusion chromatography (SEC) column (IZON, Science New Zealand). The nanopore setting was employed on the qNano, and 75 μL PBS was added. The stretch was set to 47 mm, the operating voltage was set to 0.74 V, and the pressure was set to 12 cm H_2_O. Sample size was determined via calibration with standard beads of known size.

### Fabrication of Paper-Based Immunoaffinity Devices to Capture Exosomes

The paper-based immunoaffinity devices were produced by immobilizing anti-CD63 antibodies on grade 1 Whatman filter papers (Supplementary Figure S2). Initially, the targeted reaction zone of this substrate was treated with 50 μL of 4% (v/v) 3-mercaptopropyl tri-methoxysilane (Sigma-Aldrich, United States ) in 99% ethanol (ECHO chemical, Taiwan) and incubated for 30 min. Subsequently, the reaction zone was treated with 50 μL of N-γ-maleimidobutyryloxy succin-imide ester (GMBS) (Thermo Scientific, United States ) (0.01 μmol in 99% ethanol) and incubated for 15 min. The device was then incubated in 10 μg/ml Neu-trAvidin (Thermo Scientific, United States ) solution in PBS (Thermo Scientific, United States ) at 4°C for 1 h. Blocking was conducted with 1% (w/v) bovine serum albumin (BSA) (Sigma-Aldrich, United States ) in phosphate-buffered saline (PBS) for 10 min and repeated 3 times. Finally, the reaction zone was introduced with 20 μL of biotinylated mouse anti-human anti-CD63 antibody (BioLegend, United States ) and incubated for 10 min 3 times.

### Characterization of Captured Exosomes by Field-Emission Scanning Electron Microscopy (FE-SEM)

The exosomes captured by the paper-based immunoaffinity device were treated with 20 μL of mixed paraformaldehyde (PFA) (Sigma-Aldrich, United States ) and incubated with glutaraldehyde (GA) (Sigma-Aldrich, United States ) in PBS buffer for 30 min at room temperature before being washed 3 times with PBS. In the next steps, the sampled zone was dehydrated by 20 μL of 50% ethanol in 15 min, followed by 75, 87.5, 93.75, and 99% ethanol for 10 min each. Eventually, 20 μL of 99% ethanol was applied and allowed to dry at room temperature for 30 min. The resulting samples were examined by a JSM-7500F FE-SEM (JEOL Japan).

### Quantification of Captured Exosomes by ELISA on Paper-Based Immunoaffinity Devices (P-ELISA)

Determining the exosome content by P-ELISA was accomplished by initially adding 20 μL analyte to the paper-based immunoaffinity devices and incubating for 1 h. Subsequently, the devices were treated with 5 μL of rabbit anti-human anti-CD9 antibody (1 μg/ml in PBS from Sigma-Aldrich, United States ) and incubated for 1 min before being washed 3 times with 20 µL PBS. A similar procedure of adding-incubating-washing was duplicated on the device with 5 µL of HRP-conjugated goat anti-rabbit antibody (Sigma-Aldrich, United States ). Finally, 5 μL of mixed 3,3′,5,5′-tetramethylbenzidine (TMB) (Sigma-Aldrich, United States ) and hydrogen peroxide (Sigma-Aldrich, United States ) (1:1 mix per volume) was added, and the device was incubated for color development (Supplementary Figure S3). The P-ELISA results were photographed with a cell phone camera (iPhone XR) in 8-bit format, and the color intensity from the last reaction step was quantified by ImageJ software.

### Adsorption of Exosomal Nucleic Acids by Paper-Based Silica Devices

Paper-based silica devices were prepared by adding 20 μL silica nanoparticle solution (Sigma-Aldrich, United States ) (3 mg/ml) to both sides of grade 1 Whatman filter papers before drying at room temperature for 20 min. The exosome lysate, obtained by incubating paper-based immunoaffinity devices after capturing exosomes with RNase-free water as lysis buffer at 95°C for 30 min, was then placed on paper-based silica devices at room temperature for 3 min for nucleic acid absorption (Supplementary Figure S4). Eventually, the paper-based silica devices containing attached nucleic acids were treated with RNase-free water as an elution buffer at 55°C for 45 min to collect exosomal nucleic acid solutions.

### Quantifying Exosomal miR-21 by RT-qPCR

Reverse transcription of miR-21 complementary DNA (cDNA) (by TaqMan™ MicroRNA Reverse Transcription kit from Thermo Scientific - United States ) and qPCR (by QuantiFast SYBR® green PCR kit from Qiagen—Germany; with the probes and primers shown in (Supplementary Table S1) were conducted to quantify the content of this RNA in exosomal nucleic acid solution. In detail, 10.2 μL of a solution composed of 1 μL of exosomal nucleic acid, 1 μL of 10x Poly(A)pol reaction buffer, 1 μL of 1 mM Adenosine 5′ Triphosphate, 1 μL of 1 mM deoxynucletide (dNTP) Solution Mix, 1 μL of 10 μM RT primer, 0.5 μL of Su-perScript® III reverse transcriptase, 0.2 μL of *E. coli* Poly(A) polymerase, 0.2 μL of rnase inhibitor (20 U/μL), and 4.3 μL of nuclease-free water was incubated at 42°C for 1 h before increasing the temperature to 95 °C for 5 min to inactivate the enzyme and facilitate the collection of exosomal RT cDNA. One microliter of the resulting RT cDNA, 1 μL of each primer (1.5 μM), and 5 μL of 2× QuantiFast SYBR Green PCR Master Mix (Qiagen) were dissolved in 2 μL of deionized water for qPCR, which was performed by a StepOnePlus real-time PCR system (Applied Biosystem, United States ). This process was initiated at 95 °C for 5 min, followed by 40 cycles at 95°C for 10 s and finished at 60°C for 30 s. The amplification plots and melting curves were generated using StepOne software v2.3.

### Applying the Paper-Based Kit to Capture Exosomes and Exosomal Nucleic Acids From Clinical Samples

Platelet-poor plasma (PPP) and fluidic samples of chronic lesion tissues, obtained from various patients at different times of curing progress and provided by Dr. Shin-Chen Pan—Department of Plastic Surgery, affiliated hospital of National Cheng Kung University (IRB No. B-ER-109-238), were used as clinical samples to validate the applicability of the fabricated devices. The wound fluid was directly undergone the paper-based kit without pretreatment whereas the PPP samples were separated by a sequential membrane system with pore sizes of 200 and 30 nm (Supplementary Figure S5). Theoretically, the vast majority of exosomes with a reported diameter within 30–200 nm are retained in the space between two membrane layers (Retention sample) while a minority of them can penetrate the 200-nm filtration (Filtrate sample). Exosomal miR-21 harvested from all of these clinical samples by the paper-based kit was eventually determined and evaluated via Ct values comparison. Three plasma-derived samples (PPP, Retention, and Filtrate) were also examined by Nanoparticle Tracking Analysis (NTA) as an assistance for assessing the RT-qPCR data (Supplementary Table S2).

## Results and Discussions

### Characterizing Cells Cultured in Various Microenvironments

**FIGURE 2:**
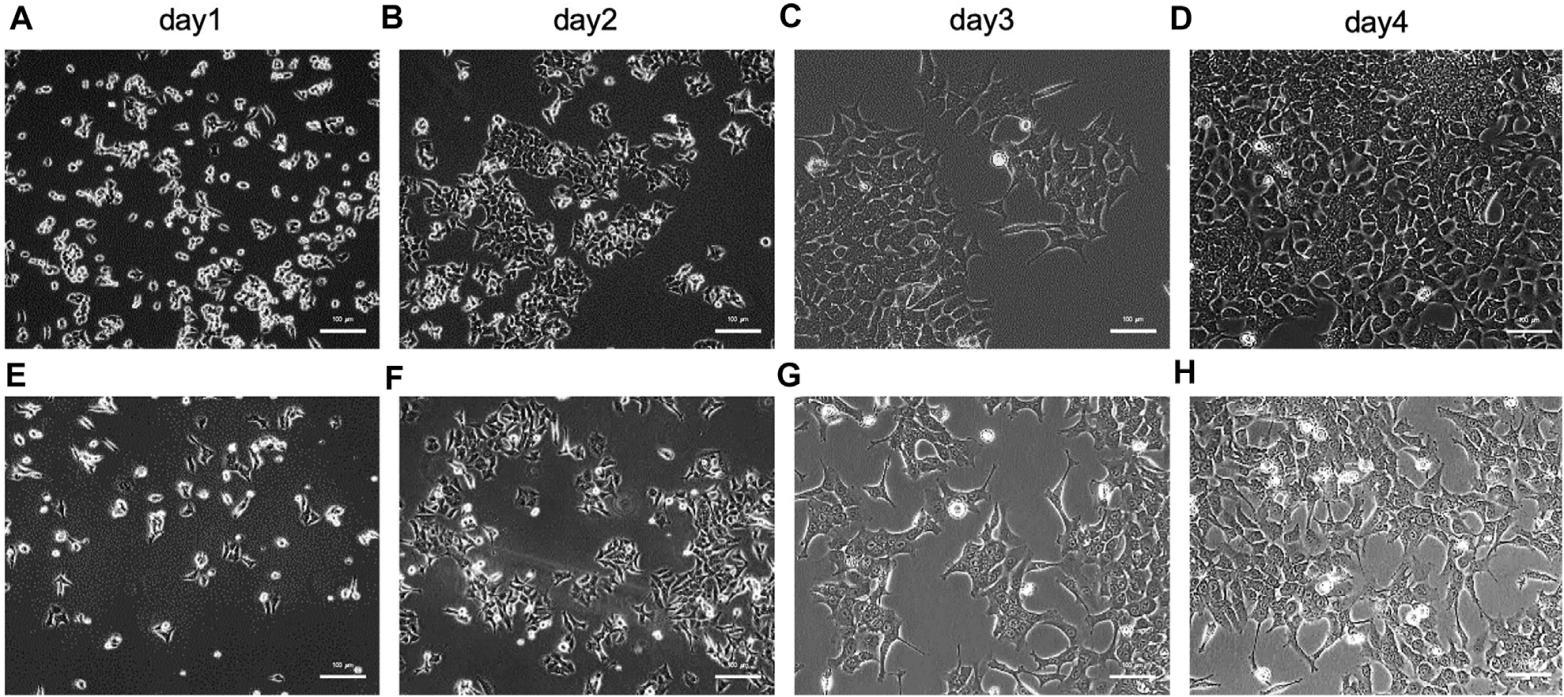
Characterization of HCT116 cells cultured in various microenvironments of **(A)**∼**(D)** pH 7.4 after **(A)** 1, **(B)** 2, **(C)** 3, and **(D)** 4 days, and **(E)**∼**(H)** pH 6.5 after **(E)** 1, **(F)** 2, **(G)** 3, and **(H)** 4 days by microscope. The scale bar is 100 µm.

The capability of paper-based devices developed in this study were examined by employing them for capturing and quantifying exosomes and exosome-derived nucleic acids from cells cultured in different microenvironments. To do this, HCT116 - a human colorectal cancer cell line - was initially cultured in pH 7.4 medium before gradually altering this parameter to 7.2, 6.9, 6.7, and 6.5. The final products from the media at pH 7.4 and 6.5 were observed by microscopy (Figure 2), which showed that there were few differences in morphology between cells cultured in microenvironments with pH 7.4 (Figures 2A–D) and 6.5 (Figures 2E–H), suggesting that it is feasible to harvest exosomes from cells cultured in various microenvironments.

### Size Distribution, Concentration, and Characteristics of HCT116 Cell-Derived Exosomes

**FIGURE 3:**
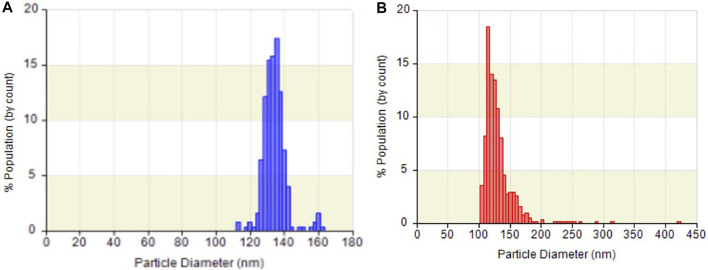
Particle size distribution and concentration of **(A)** exosomes from HCT116 cell culture medium and **(B)** commercial lyophilized exosomes analyzed by qNano.

**FIGURE 4:**
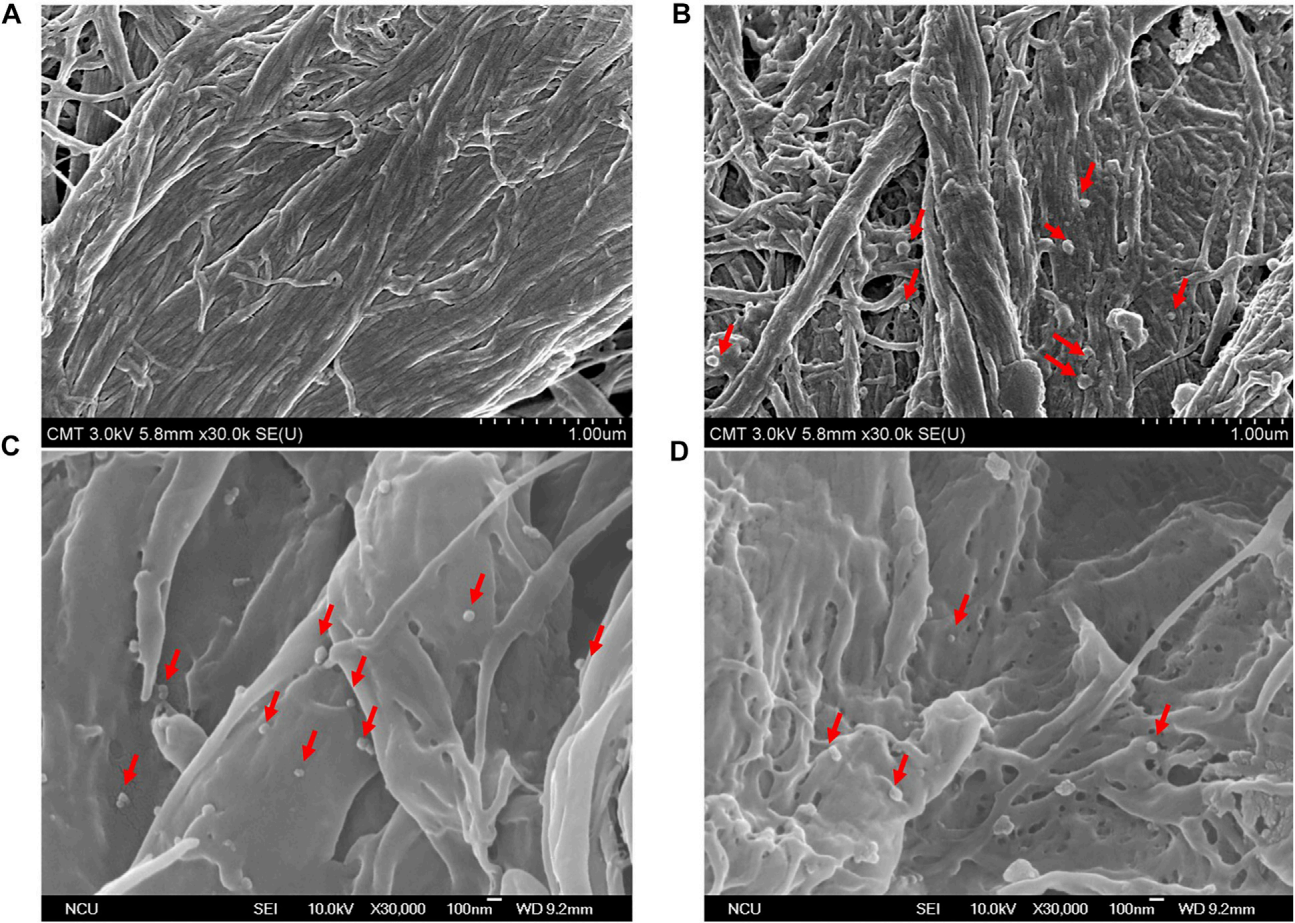
SEM pictures characterize surface morphology of **(A)** untreated sample, **(B)** commercial lyophilized exosomes, and **(C,D)** HCT116-derived exosomes captured by paper-based immunoaffinity devices. Red arrows indicate captured exosomes.

Exosomes, with a particle size of 30–150 nm encapsuled by a lipid bilayer and a concentration of approximately 10^8^ particles/mL in cell culture media (He et al., 2018; Heinemann et al., 2014), are essentially guaranteed not to be damaged during centrifugation prior to quantification by ELISA, and these exosomes were used as materials for nucleic acid extraction. Hence, the size distribution and concentration of HCT116 cell-derived exosomes in the pretreated media were statistically characterized by the qNano instrument after being ultra-filtrated, and their particle morphology was examined by FE-SEM after being captured by paper-based immunoaffinity devices. On the one hand, concentrations of 3.6×10^8^ exosomes/mL and size distributions of approximately 110–160 nm (mean diameter of 133.1 nm, Figure 3A) were observed, which was equivalent in amount and identical in size to those of the standard sample (3.4×10^9^ exosomes/mL and 100–200 nm (mean diameter of 129 nm, Figure 3B) identified from a 1.9×10^9^ exosome/mL solution of commercially available lyophilized exosomes from the HCT116 cell line (human colon carcinoma) obtained from Hansa BioMed Life Science, Estonia), indicating that HCT116 cell-derived exosomes pretreated by our proposed method met the commercial requirements. On the other hand, exosome-like particles (Enderle et al., 2015; Wu et al., 2015) appeared in the SEM images of the media isolated by the designed system (Figures 4C, D) in comparison with the SEM images of the blank sample (Figure 4A); these particles had round and cup shapes and dimensions equivalent to those of the commercial products (Figure 4B), confirming that the immunoaffinity devices fabricated in this study succeeded in capturing exosomes and therefore is applicable for further purposes.

### Quantifying Exosomes Captured on Paper-Based Immunoaffinity Devices by ELISA (P-ELISA)

**FIGURE 5:**
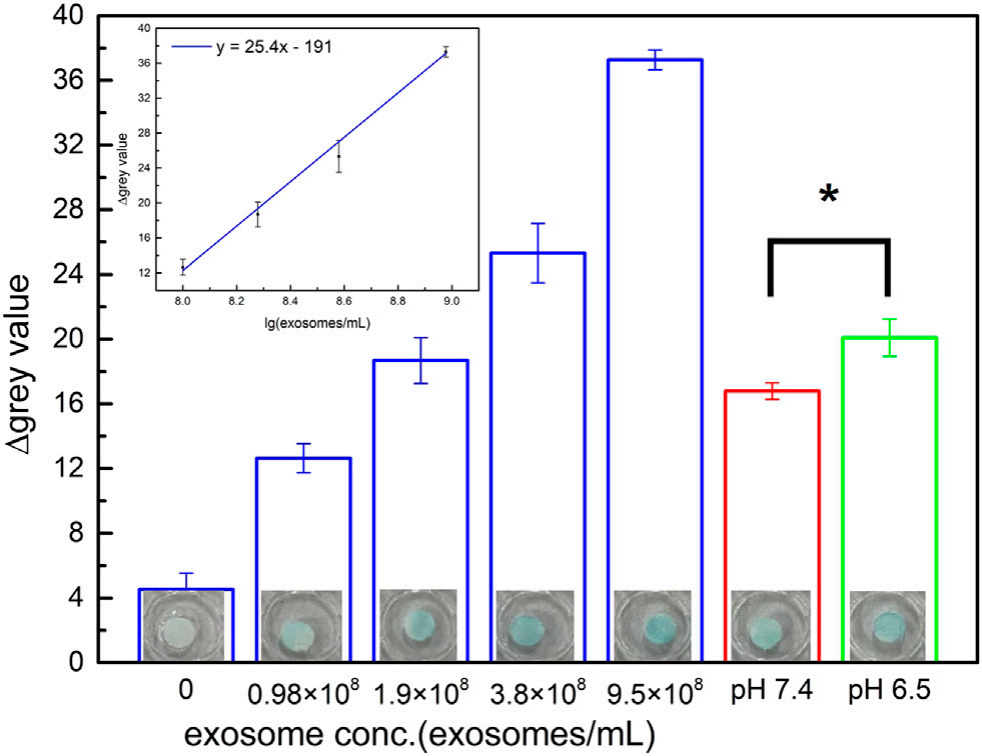
Quantification of exosomes performed by color intensity assessment including commercial lyophilized samples (blue columns) and exosomes captured from the medium of HCT116 cells cultured in various microenvironments of pH 7.4 (red column) and 6.5 (green column). Inset is the calibration curve of exosomes from commercial samples (blue columns). An asterisk (*) denotes a *p* value <0.05 (obtained from *t*-test). All of the results were obtained by P-ELISA.

In the next step, P-ELISA (described in experimental section) was used for the quantification of exosomes captured from cells cultured in the aforementioned microenvironments. The data presented in Figure 5 demonstrated that the paper-based immunoaffinity devices were capable of seizing exosomes at different contents, in which the color intensified with increasing concentrations of exosomes (Figure 5). A calibration line (y = 25.4x - 191) was therefore constructed to formulate the relationship between the exosome levels (log_10_ values, x) and the color intensity (y) (inset of Figure 5). Since the color intensities observed in the samples from the pH 7.4- and 6.5-pretreated media were statistically distinguishable (*p* value <0.05) and within the linear region of the calibration line, the exosome concentrations of these two varied microenvironments were estimated to be 1.53×10^8^ and 2.05×10^8^ exosomes/mL, respectively. These empirical figures not only indicated that cancerous cells released more exosomes in acidic microenvironments than in neutral microenvironments, which is identical to a discovery reported by several groups (Parolini et al., 2009; Logozzi et al., 2017; Boussadia et al., 2018; Logozzi et al., 2018; Tian et al., 2019), but also was an evidence proving that the designed paper-based immunoaffinity devices could effectively determine exosome amounts released in different microenvironments.

### Extracting Exosomal Nucleic Acids From HCT116 Cells Cultured in Various Microenvironments and Determining miR-21 Content by RT-qPCR

Exosomes contain a variety of molecular contents, including proteins and nucleic acids, that can be used to provide cellular information (Qing et al., 2018; Jeppesen et al., 2019; Zhang et al., 2019; Kalluri and LeBleu, 2020; Ocansey et al., 2020; Qiao et al., 2020). Paper-based silica devices can be combined with paper-based immunoaffinity devices to capture exosomal nucleic acids and have extensive applications of POCT. Herein, miR-21, a noncoding RNA of 20–24 nucleotides and among exosomal nucleic acids trapped by paper-based silica devices, was chosen as the representative target because it plays important roles in cell growth, metastasis, and chemoresistance in breast, colorectal, lung, liver, pancreatic, and prostate cancer (Bautista-Sánchez et al., 2020), such that miR-21 can be classified as a cancer “oncomiR” (He et al., 2017; Moscetti et al., 2019).

Numerous patients have been diagnosed with advanced stages of colorectal cancer, which is highly prevalent and has a high mortality rate (Tsukamoto et al., 2017). In recent years, miRNAs have been discovered to be enriched in exosomes prior to being released to body fluids (Li et al., 2015; Zhao et al., 2015). miRNAs encapsulated in exosomes are more stable in body fluids because the exosomal lipid bilayer membrane protects miRNAs from degradation by rnase (Ge et al., 2014; Bhome et al., 2017). There is evidence of significantly higher exosomal miR-21 contents in the serum of colorectal cancer patients than in that of healthy individuals, and some evidence suggests the relation of miR-21 levels to various stages of this cancer (Jin et al., 2019). Activity and concentration of miR-21, which are strongly related to tumor size, stage, and lymph node metastasis, are higher in colorectal cancerous tissues than in normal tissues and decrease in patients after completion of cancer treatment (Tsukamoto et al., 2017; Moridikia et al., 2018). These results indicate that exosomal miR-21 could be used as a biomarker for cancer diagnosis as well as extracting and detecting miR-21 may facilitate effective treatment to reduce mortality.

**FIGURE 6:**
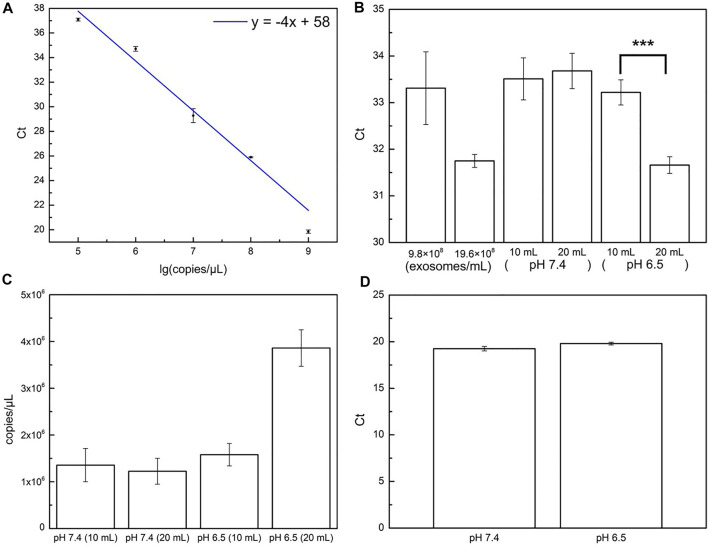
Quantification of miR-21. **(A)** Calibration curve for standard miR-21 samples by paper-based silica devices and quantified by RT-qPCR. **(B)** miR-21 contents (estimated by Ct values) in exosomes from standard samples and from 10 to 20 ml pH HCT116 samples cultured in pH 7.4 and pH 6.5 microenvironments. Three asterisks (***) denote a *p* value <0.001 (obtained from *t*-test). **(C)** miR-21 concentrations (copies/µL) of 10 and 20 ml HCT116 samples cultured in pH 7.4 and pH 6.5 microenvironments, calculated from calibration line in **(A)** and corresponding Ct values in **(B)**. **(D)** U6 SiRNA contents (estimated by Ct values) derived from HCT116 cells cultured in pH 7.4 (red column) and pH 6.5 (green column) conditions.

In this experiment, RT-qPCR was utilized to determine miR-21 and examine the ability of paper-based silica devices in trapping exosomal nucleic acids. After being treated with RNase-free lysis buffer at 95°C for 30 min, the samples were incubated with paper-based silica devices for nucleic acid adsorption before the silica-coated areas were treated with RNase-free elution buffer at 55°C water for 45 min to extract trapped nucleic acids and quantify miR-21 content by RT-qPCR. An investigation of the incubation step was implemented to identify the optimal operating time for nucleic acid adsorption. Empirical results reveal that despite equivalent Ct values (threshold cycle) obtained after the periods of 1, 3, 5, and 10 min, incubating the silica devices with the pretreated samples for 3 min provides the maximal efficiency in terms of time Supplementary Figure S4). The subsequent samples were therefore incubated with paper-based silica devices for 3 min to trap synthetic miR-21 at various concentrations to conduct a standard calibration line (Figure 6A) which was then used to calculate the miR-21 content in the exosomes from two microenvironments (Figures 6B, C). The high Ct values of 10 ml samples in the aforementioned microenvironments not only were not significantly different to each other (*p* > 0.05) (Figure 6B) but also revealed low miR-21 levels (Figure 6C), which would reduce RT-qPCR fidelity. The initial medium volumes were hence doubled to 20 ml to guarantee the analysis by RT-qPCR. As a result, the theoretical value of ΔCt (the change in Ct values between the initial volume of 10 ml and the final volume of 20 ml) should be 1. On the one hand, the estimated ΔCt of HCT116 in the pH 6.5 microenvironment was 1.56, which is similar to the 9.5 × 10 (Liu et al., 2017) exosomes/mL standard exosomes, demonstrating a significant difference compared to the 10-ml volume sample (*p* value <0.001). On the other hand, the low ΔCt (approximately 0.21) in the pH 7.4 microenvironment suggested a low RT-qPCR detection fidelity due to the small content of exosomal miR-21. The ΔCt of different initial sample volumes matches the theoretical value of miR-21 quantification by RT-qPCR, indicating that the adsorption process could sufficiently capture exosomal nucleic acids from samples of cells cultured in different media for quantification. U6 siRNA, a small RNA commonly used as a cell content control because of its intracellular origin, was used to guarantee that both exosomes and exosomal nucleic acids were secreted from equal amounts of HCT116 cells cultured in each microenvironment. U6 siRNA was amplified via RT-qPCR and TaqMan® MicroRNA assay after this nucleic acid had been extracted from the products harvested after culturing cells for 3 days in various pH conditions (following identical procedure of culturing HCT116 cells). The Ct values obtained for U6 siRNA derived from U6 cells grown in pH 6.5 and 7.4 media for 3 days were 19.80 and 19.25, respectively (Figure 6D), demonstrating that the cell contents in the two examined microenvironments were equivalent, although the amount in the pH 6.5 medium was slightly lower than that in the pH 7.4 medium because of the slightly higher Ct value in sample of pH 6.5 medium compared to that in the sample of pH 7.4 medium. A comparison between Figure 5 and Figure 6 (B and C) not only confirms that HCT116 cells cultured in an acidic environment secreted more exosomes and corresponding miR-21 but also proves that paper-based immunoaffinity and paper-based silica devices were applicable for successful and sensitive extraction.

### Extracting Exosomal miR-21 From Biological Samples by Paper-Based Devices

**FIGURE 7:**
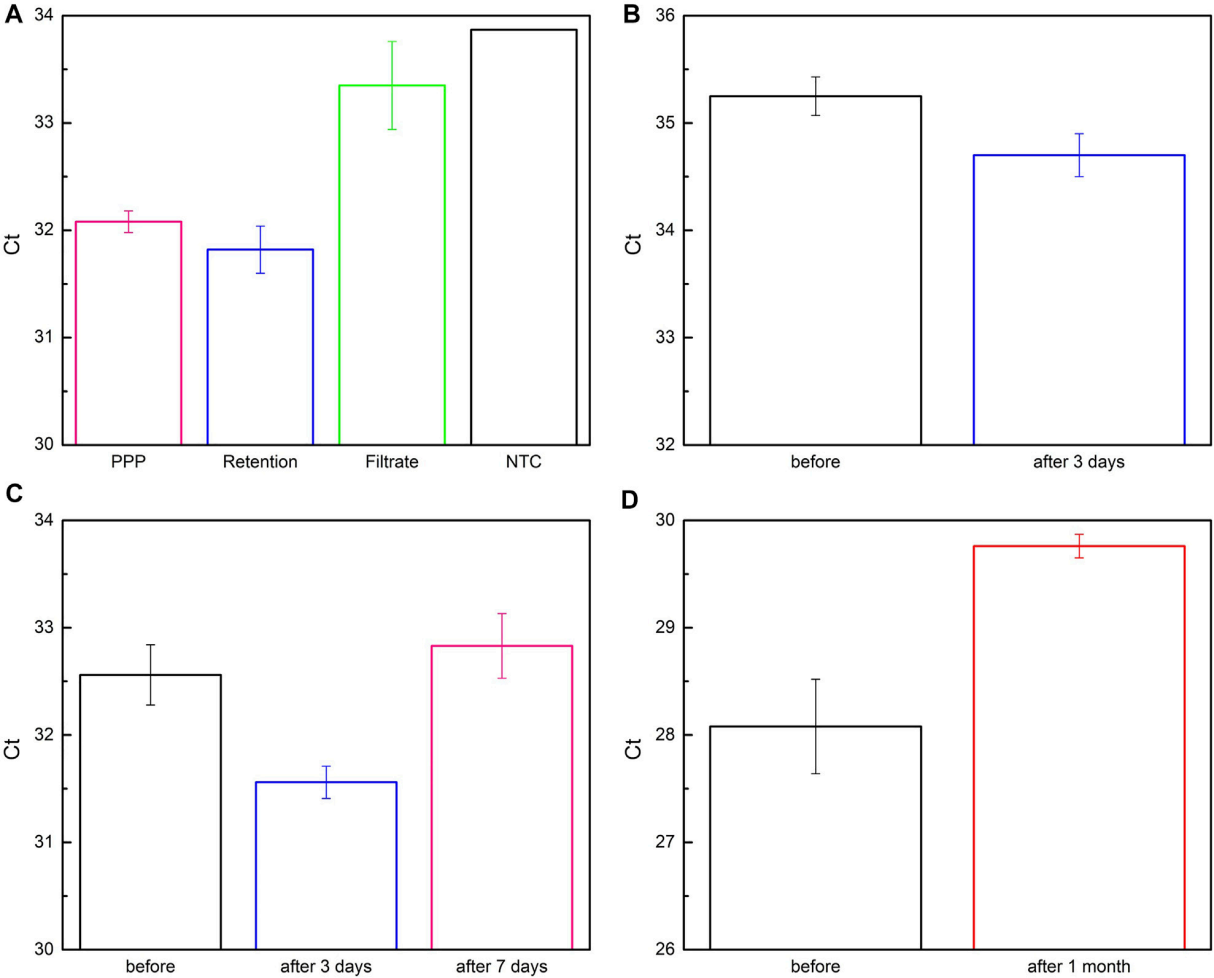
Quantification of exosomal miR-21 from bio-samples captured by paper-based system. **(A)** Plasma-derived samples were obtained by flowing platelet-poor plasma (PPP, pink column) through 200 nm- and 30 nm-membrane to collect Retention (blue column) and Filtrate (green column) samples, respectively. No template control (NTC) was for comparison and analysis. **(B–D)** Fluidic samples were obtained from lesions of patients before and after being monitored for **(B)** 3 days, **(C)**, 7 days, and **(D)** 1 month.

In this section, plasma and wound fluid were used as biological samples to evaluate the clinical applicability of the deployed system. Three plasma-derived samples (PPP, Retention, and Filtrate), formed after filtering PPP with two membranes, were applied on paper-based immunoaffinity and paper-based silica devices for nucleic acid capture (Supplementary Figure S5). Their exosomal miR-21 contents were then analyzed by qRT-qPCR and compared with no template control (NTC) sample (Figure 7A). Evidently, Ct value achieved from Filtrate was remarkably higher than others and close to that value of NTC, indicating that exosomes are scarcely present in Filtrate. On the other hand, there was only a slight difference between the Ct values of Retention and PPP, suggesting an approximately equal amount of exosomal miR-21 extracted, which is also consistent with the output by NTA in terms of exosome concentrations (Supplementary Table S2). These results are, on one hand, reasonable in the context of the possible presence of undesirable molecules of the same sizes as well as exosome loss during filtration that cause NTA measurement error. On the other hand, low exosomal miR-21 counts (high Ct values) in spite of abundant exosomes are congruent with the samples from healthy plasma. It is also important to note that the working yield of the paper-based kit in the PPP sample was comparable with the Retention sample (inferred from their equivalent Ct values), even though the PPP was not pre-filtered by two layers of the membrane system to eliminate the interferences from the particles with dimensions outside the range of exosomes. These results indicate that the developed paper-based kit is feasibly combined with sequential membranes for capturing exosomes and exosomal nucleic acids from complex clinical bio-samples.

Encouraged by successful trials with PPP under the facilitation of a sequential membrane system, we furtherly assessed the aptitude of two paper-based devices without assistance by applying them for wound fluid, a more complicated sample than PPP with cells, red blood cells, bacteria, etc. Fluidic samples secreted from chronic lesions before and after 3-day, 7-day, and 1-month surveillance were collected and investigated by comparing the Ct values of miR-21 after RT-qPCR (Figures 7B–D), since miR-21 has been reported to be greatly involved in wound healing process (Herter and Xu Landén, 2017). It is noteworthy that exosomal miR-21 expression is distinctly elevated in preliminary phase of the wounds (3 days in Figures 7B, C) compared to the longer periods (7 days in Figures 7C, 1 month in Figure 7D). Its content started reducing after 7 days (Figure 7C) and considerably declined after 1 month (Figure 7D) when the lesions had been widely swollen and ulcerated. This tendency implied a relationship between exosomal miR-21 and the severity of the lesions as well as raised a hypothesis that exosomal miR-21 not only promotes wound healing but also serves as a biomarker for monitoring and predicting treatment efficacy. These observations were in good agreement with previous investigations, indicating that miR-21 plays a role in healing wound as documented in the literature (Herter and Xu Landén, 2017). In short, these results demonstrated that the paper-based kit was competent for capturing exosomes and exosomal miR-21 in various types of biological samples.

## Conclusion

In this study, the designed paper-based system, comprising of paper-based immunoaffinity devices and paper-based silica devices, has successfully captured exosomes and exosomal nucleic acids from a variety of biological samples with easy operation and high efficiency. The paper-based immunoaffinity devices proved that there were more exosomes released by HCT116 cells cultured under acidic conditions than by HCT116 cells cultured under neutral conditions, which is correspondent with higher levels of miR-21 trapped by the paper-based silica devices. The developed kit also succeeded in isolating exosomes and exosomal nucleic acids from PPP and lesion fluids as clinical applications. They can be used alone or in association with another filtration technique. This approach with thin papers therefore not only provides a novel method for capturing exosomes and nucleic acids without requiring expensive equipment, chemicals, extensive training or professional support but also holds great potential to integrate into POCT applications, especially in areas or countries with low resources.

## Data Availability

The raw data supporting the conclusion of this article will be made available by the authors, without undue reservation.
