# Supplementary material for: Paper-Based Devices for Capturing Exosomes and Exosomal Nucleic Acids From Biological Samples
Source: Front Bioeng Biotechnol. 2022 Apr 12;10:836082. doi: 10.3389/fbioe.2022.836082 (PMC9039228; doi:10.3389/fbioe.2022.836082)
Supplement: Supplementary file 1 [file DataSheet1.docx]

***Supplementary Material for***

Paper-based devices for capturing exosomes and exosomal nucleic acids from biological samples

**Chi-Hung Lai^1^, Chih-Ling Lee^1^, Cao-An Vu^1^, Van-Truc Vu^1^, Yao-Hung Tsai^2^, Wen-Yih Chen^1,^*, Chao-Min Cheng^2,^***

^1^ Department of Chemical and Materials Engineering, National Central University, Taoyuan 32001, Taiwan

^2^ Institute of Biomedical Engineering, National Tsing Hua University, Hsinchu 30003, Taiwan.

* Corresponding author:

Wen-Yih Chen, Tel: +886-3-422-7151#34222, Email address: wychen@ncu.edu.tw

Chao-Min Cheng, Tel: +886-3-5715131 #62420, Email address: chaomin@mx.nthu.edu.tw


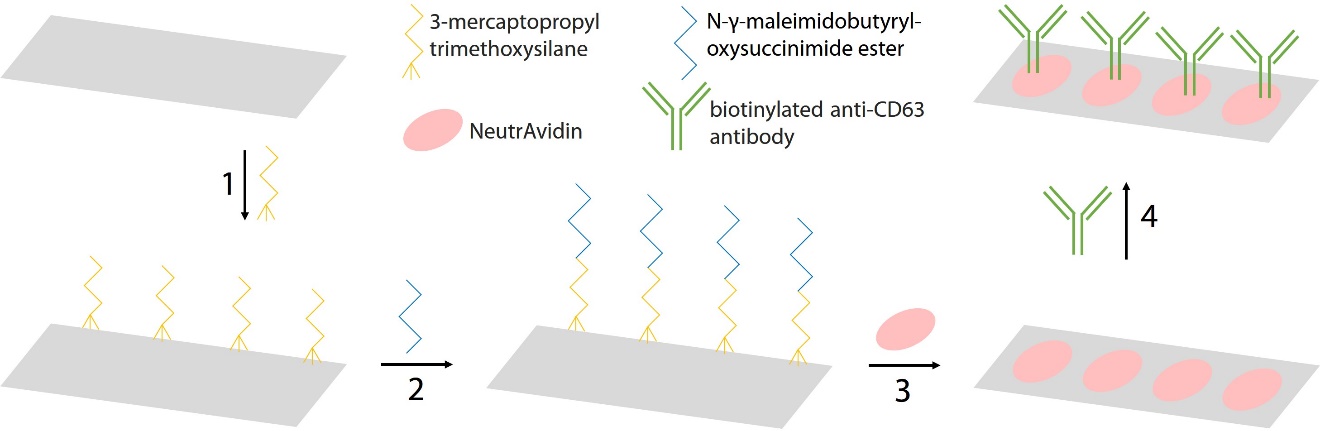


**Supplementary Figure 1**. Fabrication of paper-based immunoaffinity devices


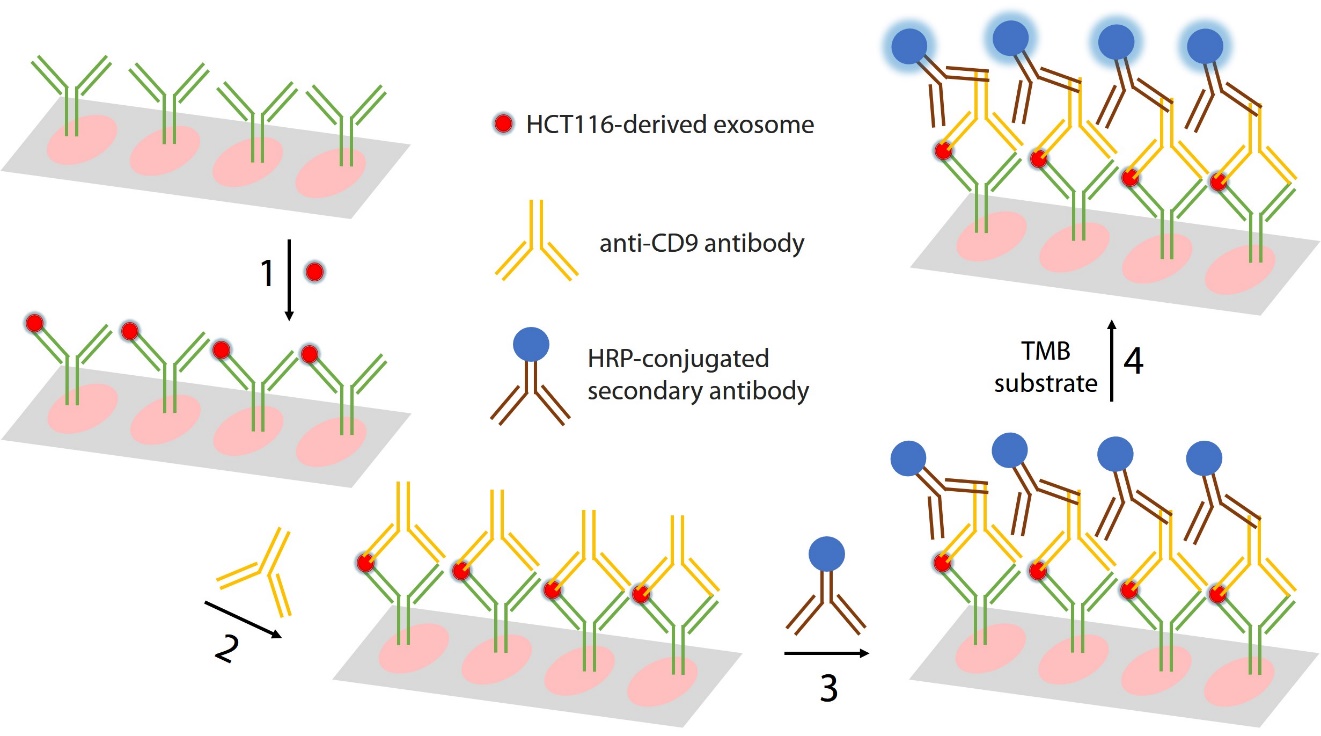


**Supplementary Figure 2**. Experimental procedure of quantifying captured exosomes by enzyme-linked immunosorbent assays (ELISA) on paper-based devices (P-ELISA)


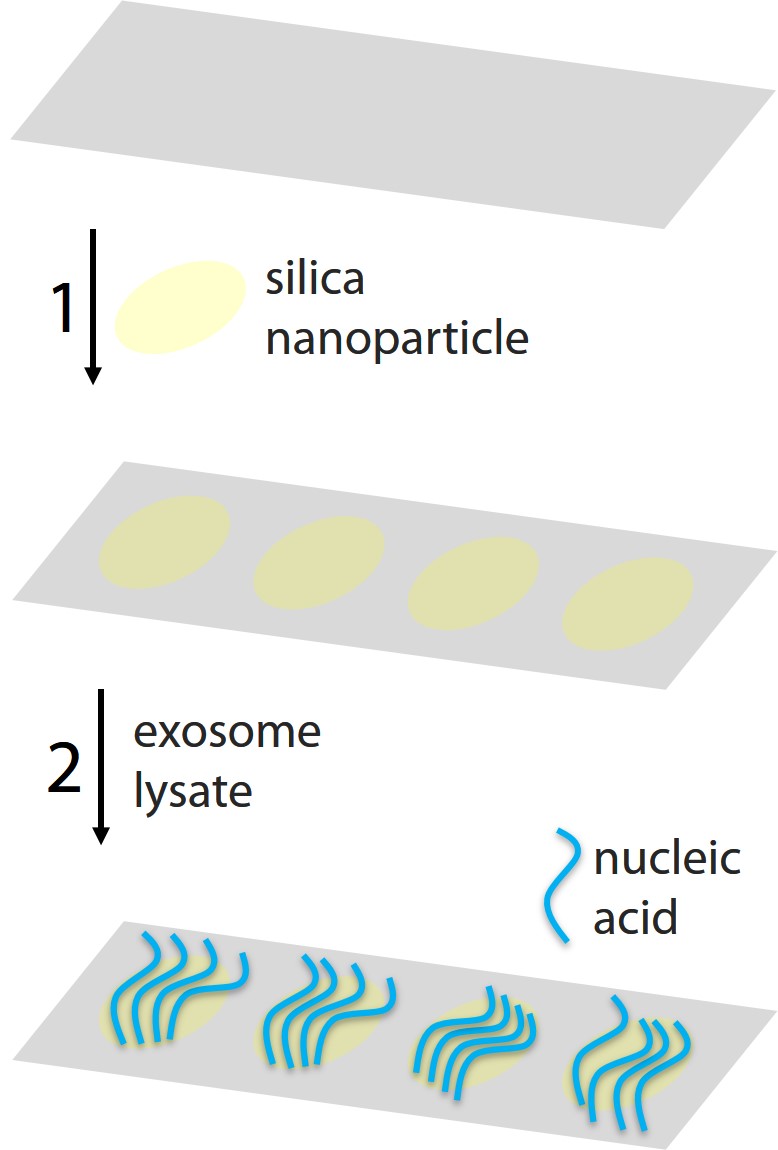


**Supplementary Figure 3**. Fabrication of paper-based silica devices


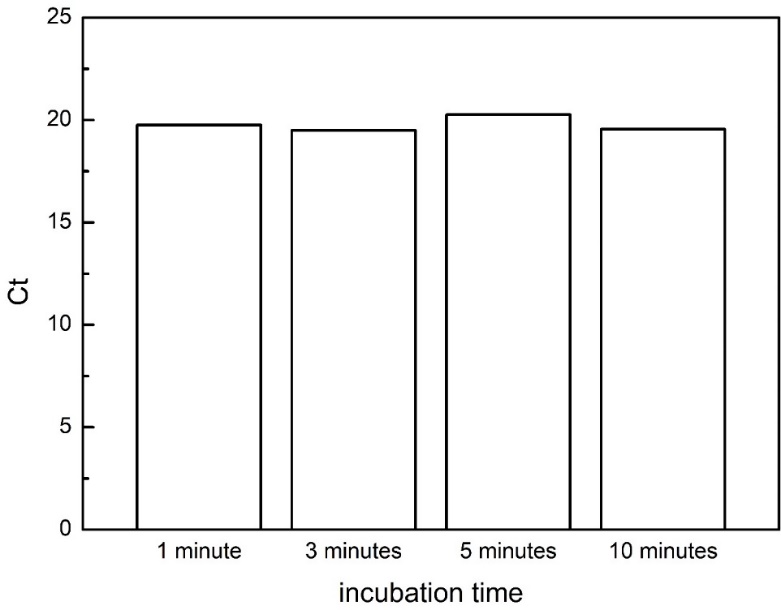


**Supplementary Figure 4**. Different incubation periods for miR-21 with paper-based silica devices


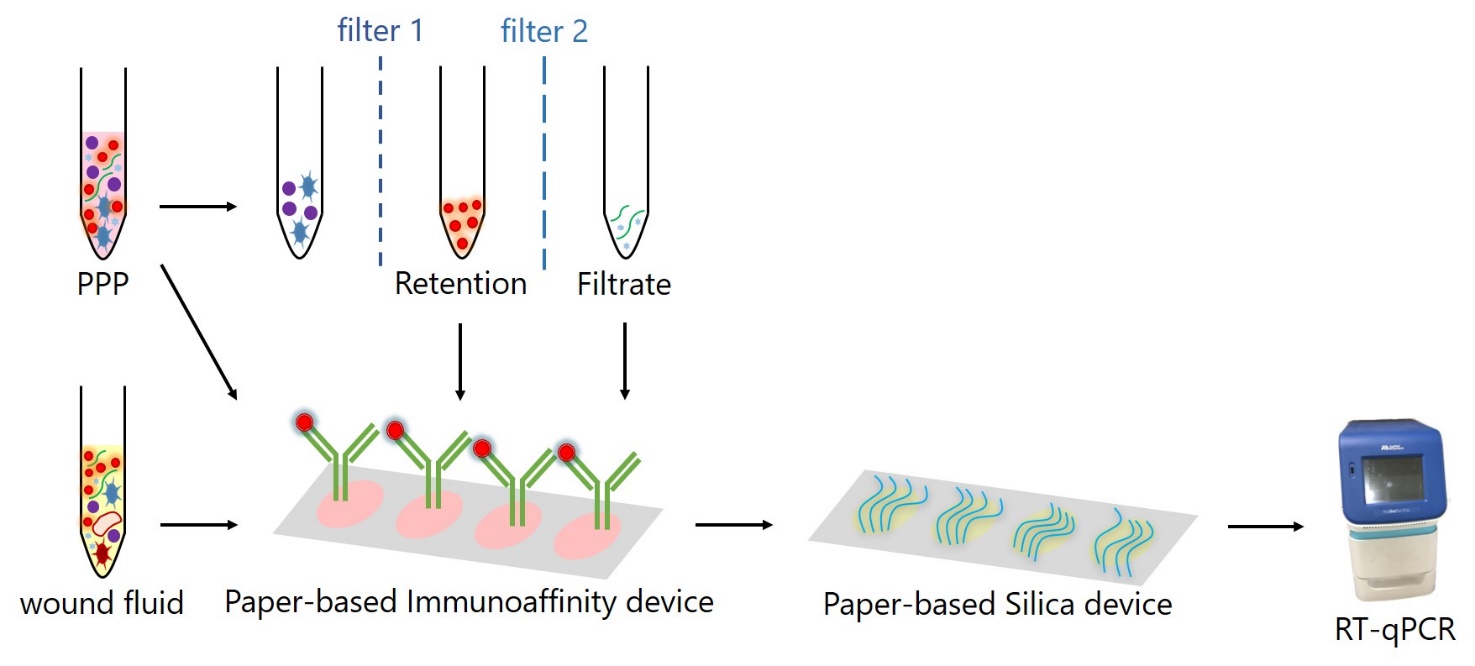


**Supplementary Figure 5.** The procedure of capturing exosomes and exosomal nucleic acids from biological samples (plasma-derived samples and wound fluid)

Table S1. Probes and primers for reverse transcription and RT-qPCR

| Probes and Primers | Sequences |
| --- | --- |
| miRNA-21 | 5’-UAGCU UAUCA GACUG AUGUU GA-3’ |
| universal reverse transcription primer | 5’-CAGGT CCAGT TTTTT TTTTT TTTTV N-3’ |
| miRNA-21 forward primer | 5’- TCAGT AGCTT ATCAG ACTGA TG-3’ |
| miRNA-21 reverse primer | 5’-CGTCC AGTTT TTTTT TTTTT TTCAA C-3’ |

Table S2. Exosome concentrations examined by nanoparticle tracking analysis in three plasma-derived samples.

| Sample | Concentration (10^9^ particles/mL) | Error |
| --- | --- | --- |
| Platelet-poor plasma (PPP) | 770 | 29.4 |
| Retention | 685 | 28.9 |
| Filtrate | 1.67 | 0.07 |
